# Molecular and epigenetic responses to crowding stress in rainbow trout (*Oncorhynchus mykiss*) skeletal muscle

**DOI:** 10.3389/fendo.2025.1571111

**Published:** 2025-04-16

**Authors:** Daniela Aravena-Canales, Valentina Valenzuela-Muñoz, Cristian Gallardo-Escarate, Alfredo Molina, Juan Antonio Valdés

**Affiliations:** ^1^ Laboratorio de Biotecnología Molecular, Facultad de Ciencias de la Vida, Universidad Andrés Bello, Santiago, Chile; ^2^ Interdisciplinary Center for Aquaculture Research (INCAR), University of Concepción, Concepcion, Chile; ^3^ Laboratory of Biotechnology and Aquatic Genomics, Department of Oceanography, University of Concepción, Concepcion, Chile; ^4^ Centro de Investigación Marina Quintay (CIMARQ), Universidad Andrés Bello, Quintay, Chile

**Keywords:** oxidative stress, aquaculture, methylome, transcriptome, growth

## Abstract

**Background:**

Chronic stress is a critical challenge in fish aquaculture, adversely affecting growth, health, and overall productivity. Among the most significant chronic stressors in intensive farming is crowding, which triggers the release of cortisol, the primary stress hormone in fish. Cortisol re-allocates energy away from growth-related processes toward stress response mechanisms. Consequently, overcrowded fish often exhibit slower growth rates, and impaired skeletal muscle development. Understanding the mechanisms underlying crowding stress and their long-term effects, including epigenetic changes, is essential for optimizing farming conditions, and enhancing fish welfare.

**Objective:**

This study aims to characterize the physiological, transcriptomic, and epigenomic responses in juvenile rainbow trout (*Oncorhynchus mykiss*) exposed for 30 days to high stocking densities.

**Results:**

Crowding stress led to decreased weight in the high-density (HD) group. It also resulted in elevated cortisol levels, oxidative DNA damage, and protein carbonylation in skeletal muscle. Using RNA-seq, we identified 4,050 differentially expressed genes (DEGs), and through whole-genome bisulfite sequencing (WGBS), we detected 11,672 differentially methylated genes (DMGs). Integrative analyses revealed 263 genes with a negative correlation between upregulated expression and downregulated methylation, primarily associated with autophagy, mitophagy, and the insulin signaling pathway. Conversely, 299 genes exhibited the reverse trend, mainly linked to ATP-dependent chromatin remodeling.

**Conclusion:**

This study offers the first detailed exploration of the molecular responses in skeletal muscle to crowding stress, integrating RNA-seq and WGBS analysis in rainbow trout, offering valuable information for improving aquaculture practices.

## 1 Introduction

Fish aquaculture plays a vital role in meeting the growing global demand for seafood, contributing significantly to food security, economic development, and environmental sustainability (1). Salmonid farming is among the most important sectors of fish aquaculture, making a substantial contribution to global aquatic food production (2). While Atlantic salmon (*Salmo salar*) dominates the global market, Rainbow trout (*Oncorhynchus mykiss*) farming is a critical component of salmonid aquaculture, particularly in freshwater systems (3). Renowned for its adaptability, rapid growth, and nutritional value, rainbow trout is cultivated in diverse environments supporting both small- and large-scale production (4, 5). In 2022, inland and coastal aquaculture of rainbow trout reached a volume of 959,600 tonnes, valued at USD 3.2 billion (6). Despite advances in aquaculture, intensive systems often induce stress, harming fish health, growth, and welfare (7). Common stressors include poor water quality, overcrowding, handling during transport or harvest, temperature fluctuations, and disease outbreaks (8). These challenges trigger physiological stress responses, most notably the release of cortisol, the primary stress hormone in fish (9). Chronic stress impairs both innate and adaptive immune responses, increasing the susceptibility to diseases (10). Chronic stress significantly affects the psychological and ethological condition of fish, impacting their behavior and social interactions (11, 12). It disrupts metabolism, diverting energy from growth to survival, reducing feed intake, and accelerating muscle loss (13, 14). In addition, Chronic stress weakens muscle fibers, slowing growth, reducing weight, and impairing swimming (15–19).

Chronic stress in fish can induce significant oxidative stress in skeletal muscle, adversely affecting cellular health, growth, and overall fish quality (20). Prolonged stress elevates cortisol levels, which increases metabolic rates and leads to production of ROS (reactive oxygen species). These ROS can damage critical cellular components, including lipids and DNA (21). Oxidative stress also promotes protein carbonylation, altering protein structure and disrupting essential muscle functions such as metabolism and contraction efficiency (22). Moreover, chronic stress may suppress the expression of antioxidant enzymes, rendering muscle tissue more susceptible to sustained oxidative damage (23, 24). A recent study on Rainbow trout demonstrated that chronic stress from high stocking densities elevates cortisol levels, disrupting the balance of muscle protein turnover. This imbalance affects three critical proteolytic pathways: the UPS (ubiquitin-proteasome system), the ALS (autophagy-lysosome system), and the calpain system (25). These pathways, essential for maintaining muscle integrity and supporting growth, become dysregulated under chronic stress, resulting in muscle degradation, and reduced growth rate (25). Similarly, a study on Fine flounder (*Paralichthys adspersus*) found that crowding stress elevated cortisol levels, disrupting growth-promoting pathways such as the growth hormone/insulin-like growth factor axis. This stress also activated muscle breakdown mechanisms through the ubiquitin-proteasome system and autophagy, further inhibiting growth (26).

Chronic stress is increasingly recognized for its influence on epigenetic memory in fish, affecting not only immediate physiological responses but also long-term gene ex-pression patterns (27, 28). Epigenetic modifications, such as DNA methylation and histone modifications, serve as mechanisms through which environmental stressors, including chronic stress, leave lasting imprints on the genome without modifying the fundamental DNA sequence (29). These stress-induced epigenetic changes can impact key biological processes, including growth, immune function, metabolism, and reproductive capacity. Furthermore, they may influence not only the stressed individual but also subsequent generations (27). By modulating gene expression in a long-lasting manner, epigenetic memory enables fish to adapt to recurring stressors. However, it can also result in maladaptive outcomes, such as reduced growth efficiency or heightened disease susceptibility. Although there are no studies specifically examining the effects of crowding on growth in aquaculture-relevant species, its epigenetic impacts have been studied in zebrafish (*Danio rerio*). Recent research revealed that maintaining zebrafish larvae at high densities led to changes in the methylation of promoter regions associated with DNA maintenance methylation, reproduction, and stress response (30). Similarly, in adult male zebrafish skin, a global reduction in DNA methylation levels was observed, particularly in processes linked to immune response and oxidative stress (31). Understanding how chronic stress shapes epigenetic memory in fish is essential for improving fish health and welfare in aquaculture. This knowledge paves the way for developing strategies to mitigate the long-term negative effects of stress. As sentient beings, fish experience pain and stress in ways comparable to mammals, making their welfare a critical consideration in aquaculture and research practices. In the present study, we examined the effects of crowding on juvenile Rainbow trout reared at high stocking densities using RNA-Seq and whole-genome bisulfite sequencing (WGBS). This study aims to provide a comprehensive analysis of the physiological and molecular impacts of crowding stress in rainbow trout, with a focus on the potential long-term negative effects of these farming practices. We identified numerous differentially expressed and methylated genes, many of which were associated with critical processes involved in skeletal muscle catabolism and atrophy, including autophagy/mitophagy, insulin signaling pathway, and ATP-dependent chromatin remodeling.

## 2 Methodology

### 2.1 Protocol experiment

This study followed bioethical protocol authorized by the ethical committee of the Universidad Andres Bello (protocol code 010/2023). Juvenile Rainbow trout (*O. mykiss*) (7.24 ± 1.35 g) were obtained from Pisciculture Rio Blanco (PUCV, Chile) and randomly assigned to six 16 L tanks with aerated, dechlorinated water at 15 ± 1°C. Four tanks contained 10 individuals, corresponding to low-density group (LD) (control condition) (10 kg/m^3^), and four tanks contained 30 individuals, corresponding to high-density condition (HD) (experimental condition) (30 kg/m³). Experiments were carried out in 100-cm high tanks with water turnover rate of 3.5 l/min ensuring an oxygen concentration of 7 mg/l. The Rainbow trout were fed daily with salmon pellets at 1.5% of their body weight under a 12:12 h light-dark cycle. In all groups it was verified that food consumption was the same. Fish from LD and HD groups were sampled at 30 days of crowding. Six fish per tank were randomly euthanized under anesthesia (benzocaine 300 mg/l). Time sampling and rearing density were defined based on the experience and results obtained in Valenzuela et al. (25). Blood from caudal vessel was collected using 1 mL heparinized (10 mg/mL) syringe, then centrifuged at 2000 x g for 5 min at 4°C to collect plasma, immediately stored at − 80°C. Myotome skeletal muscle was sampled from all fish, specifically a cross section from the epaxial area. Finally, samples were frozen by liquid nitrogen and stored at − 80°C until processed for RNA and DNA extraction.

### 2.2 Growth performance, physiological, and oxidative stress parameters

On day 0 and 30 of the trial, body weight (g) and total length (cm) of each sampled fish were measured. The condition factor (K) was calculated as K= (W/l^3^) x 100, where W is body weight and l is the total body length. The plasmatic levels of cortisol, creatine kinase activity, and glucose, were quantified using the Cortisol ELISA Kit (Cayman Chemical, Ann Arbor, MI, USA), Creatine Kinase Assay Kit (Abcam, Cambridge, UK), and Glucose Assay Kit (Abcam, Cambridge, UK), respectively. DNA oxidative damage, protein carbonylation, and lipid peroxidation were determined using OxiSelect Oxidative DNA Damage Quantification (Cell Biolabs, San Diego, CA, USA), OxiSelect Protein Carbonyl Spectrophotometric Assay (Cell Biolabs, San Diego, CA, USA), and OxiSelect HNE Adduct Competitive ELISA Kit (Cell Biolabs, San Diego, CA, USA) respectively. Catalase (CAT) and superoxide dismutase (SOD) activities were quantified using the Abcam catalase (Abcam, Cambridge, UK) and superoxide dismutase Activity Assay Kit (Abcam, Cambridge, UK), respectively. The use of this methodology in fish tissue has been previously validated (32). The parameters measured and the catalog code used are summarized in the **Supplementary Table S1**.

### 2.3 RNA extraction, RNA sequencing, and transcriptome analysis

Total RNA was extracted from Rainbow trout skeletal muscle of the LD and HD groups. RNA extraction was performed using the Trizol Reagent (Ambion, Carlsbad, CA, USA), following the manufacturer’s instructions. RNA integrity was confirmed by electrophoresis in agarose gel (1.2%). The isolated RNA’s quality, purity, and quantity were measured in TapeStation 2200 (Agilent Technologies Inc., Santa Clara, CA, USA). Three individuals from the LD group and three individuals from the HD group were randomly selected, for cDNA libraries construction. cDNA libraries were generated using the TruSeq RNA Sample Preparation kit v2 (Illumina, USA). NGS sequencing was performed by the Macrogen Inc. (Seoul, Korea) company on the Novaseq Illumina platform. Using a paired-end technique (2 x101 bp). Raw reads were trimmed removing sequences of low quality (Q20) and those less than 40 pb. Trimmed reads were mapped onto the reference rainbow trout genome USDA_OmykA_1.1 (RefSeq GCF_013265735.2) using CLC genomic workbench software v23 (CLC bio - Qiagen, TX, USA). The settings were minimum length fraction = 0.8 and minimum similarity fraction (long reads) = 0.8; the expression value was established as transcripts per million reads (TPM). The distance metric was calculated with the Manhattan method, and Kal’s test was used to compare gene expression levels in fold change (P = 0.0005, FDR corrected). Differential expression analysis was conducted for tissue samples and filtered by absolute fold-change values ≥ 2 and FDR corrected p-value < 0.05. The identification of GO and KEGG enrichment analysis of DEGs was previously described (33).

### 2.4 DNA extraction, WGBS sequencing, and methylome analysis

Genomic DNA was extracted using DNeasy Blood & Tissue Kit (Qiagen, TX, USA) following the manufacturer’s recommendations. DNA concentration and integrity were determined by Qubit dsDNA BR Assay (Invitrogen, CA, USA) and agarose Gel Electrophoresis, respectively. Genomic DNAs were fragmented into 100-300 bp by Sonication (Covaris, Massachusetts, USA) followed by bisulfite processing. 6 DNA library were prepared by Zymo-Seq WGBS Library Kit (ZymoResearch, USA), using 100 ng of input DNA. Sequencing was performed using a paired-end strategy (2 x 150 bp) with the Novaseq (Illumina) platform of Macrogen (Seul, Korea). Raw reads were trimmed removing sequences of low quality (Q20) and those less than 40 pb. Trimmed reads were analyzed using the Bisulfite sequencing tools on using CLC genomic workbench software v23 (CLC bio-Qiagen, TX, USA). Bisulfite data were mapped to Rainbow trout genome USDA_OmykA_1.1 (RefSeq GCF_013265735.2) to locate the CG, CHG, and CHH markers. The methylation levels were determined with the tool Call Methylation levels of CLC Genomics Workbench v23 (CLC bio-Qiagen, TX, USA). Finally, the differentially methylated nearby genes were extracted from the O. mykiss genome using the CLC Genomics Workbench v23 (CLC bio-Qiagen, TX, USA). The identification of GO and KEGG enrichment analysis of DMGs was previously described (34).

### 2.5 RT- qPCR validation

RNA extraction was performed using the Trizol Reagent (Ambion, Carlsbad, CA, USA), following the manufacturer’s instructions. RNA integrity was confirmed by electrophoresis in agarose gel (1.2%). The RNA was quantified using NanoDrop technology (BioTek Instruments, VT, USA). For cDNA synthesis, 1 μg of RNA was reverse transcribed into cDNA using 5X All-In-One RT MasterMix (Applied Biological Materials Inc., Richmond, BC, Canada) under manufacturer recommendations. The real-time PCR (qPCR) was performed in a MX3000P thermocycler (Agilent Technologies, CA, USA), following the previously described protocol (35). Relative expression level of mRNA was calculated using the 2−ΔΔCt method. Relative expression analysis was calculated using geNorm software (https://genorm.cmgg.be/), and the results are expressed as the fold induction compared with control group and using beta-actin (actβ) and 40S ribosomal protein S30 (fau) as housekeeping genes. We used Primer3 software (36) for primer design (**Supplementary Table S2**).

### 2.6 Statistical analysis

Growth performance data were analyzed by the two-way ANOVA followed by Tukey’s multiple range tests. Physiological and oxidative stress data were analyzed by a non-parametric Student’s t-test. Data was presented as average ± standard error, and the statistical significance was set at P < 0.05. Data for RT-qPCR validation were analyzed by one-way ANOVA and Tukey’s honestly significant difference as a *post hoc* test. Correlation analysis between RNA-seq and Q-RT-PCR was performed using Pearson’s correlation coefficient. All statistical analyses were performed with Graph Prism 7.0 software (GraphPad Software, Inc., San Diego, CA).

## 3 Results

### 3.1 Physiological analysis of crowding stress

**Figure 1:**
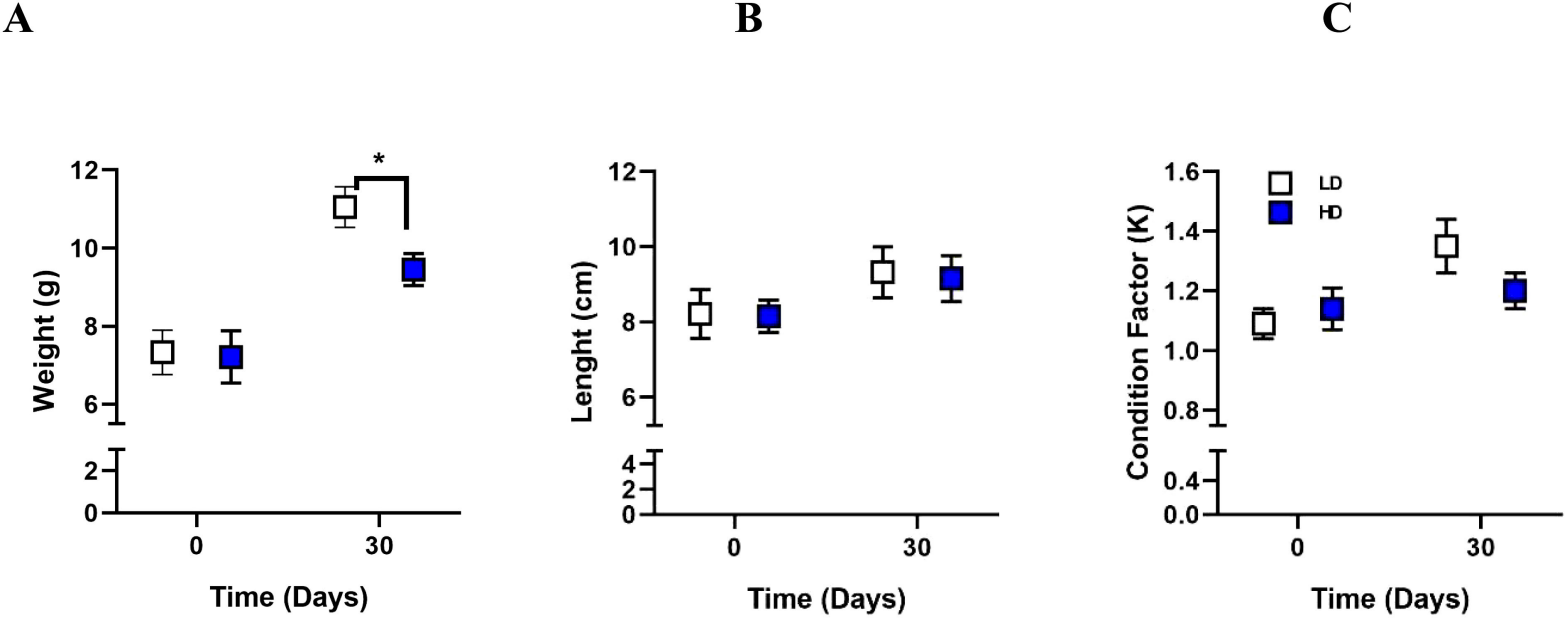
Assessment of growth performance to crowding. Weight **(A)**, length **(B)**, and condition factor K **(C)** were assessed in Rainbow trout at 30 days under Low Density (LD) and High Density **(HD)** conditions. The results are expressed as means ± standard error (n = 4). Differences between LD and HD groups are shown in *p < 0.05.

To evaluate the effects of crowding on Rainbow trout growth performance, total weight (g), body length (cm), and condition factor (K) were measured. After 30 days of crowding stress, the high-density (HD) group exhibited significantly lower weight (p-value = 0.0285) (**Figure 1A**) compared to the low-density (LD) group. No significant differences in length (**Figure 1B**) were observed between the HD and LD groups at 30 days crowding stress (p-value = 0.9970). No significant differences in condition factor (p-value = 0.9207) (**Figure 1C**) were observed between the HD and LD groups.

**Figure 2:**
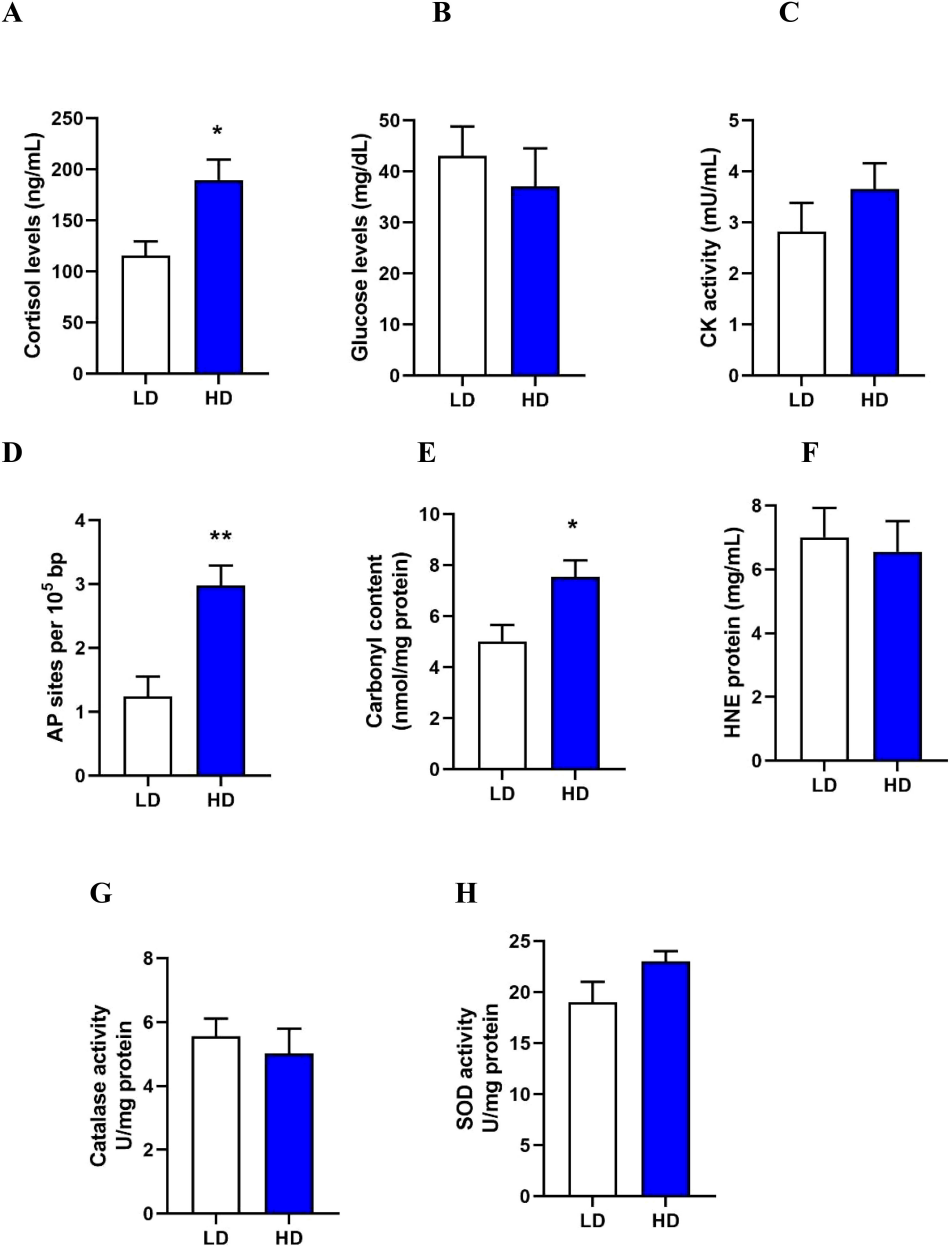
Assessment of stress response to crowding **(A-C)**. Cortisol **(A)**, glucose **(B)**, and creatine kinase activity **(C)** in plasma. Evaluation of oxidative damage in the skeletal muscle to crowding **(D-F)**. DNA oxidation as AP sites **(D)**, protein carbonylation **(E)**, and lipid peroxidation as HNE adducts **(F)**. Evaluation of antioxidant activity in the skeletal muscle to crowding **(G-H)**. Level of catalase activity **(G)**, and superoxide dismutase (SOD) activity **(H)**. All measurement were assessed in rainbow trout at 30 days under Low Density (LD) and High Density (HD) conditions. The results are expressed as a means + standard error (n= 4). Differences between LD and HD groups are shown in *p < 0.05, **p < 0.01.

To assess stress levels in the LD and HD groups, plasma cortisol, glucose, and creatine kinase (CK) activity were measured. After 30 days, cortisol levels (p-value = 0.0231) (**Figure 2A**) were significantly higher in the HD group compared to the LD group. No significant differences in plasma glucose levels (p-value = 0.5502) (**Figure 2B**) and CK activity (p-value = 0.3152) (**Figure 2C**).

To determine the impact of crowding on oxidative stress in the skeletal muscle of Rainbow trout, markers of oxidative damage, including DNA oxidation, protein carbonylation, and lipid peroxidation, were evaluated. Crowding stress caused oxidative stress in the skeletal muscle, evidenced by a significant increase in DNA oxidation (p-value = 0.0074) (**Figure 2D**) and protein carbonylation (p-value = 0.0318) (**Figure 2E**) in the HD group after 30 days of stress. No significant differences were observed in lipid peroxidation (p-value = 0.7452) (**Figure 2F**).

To assess the antioxidant response to crowding stress, the activities of catalase and superoxide dismutase (SOD) in skeletal muscle were measured. No significant differences in catalase activity (p-value = 0.5854) (**Figure 2G**) and SOD activity (p-value = 0.1238) (**Figure 2H**) were detected at 30 days of crowding stress.

### 3.2 Transcriptomic analysis of crowding stress

**Figure 3:**
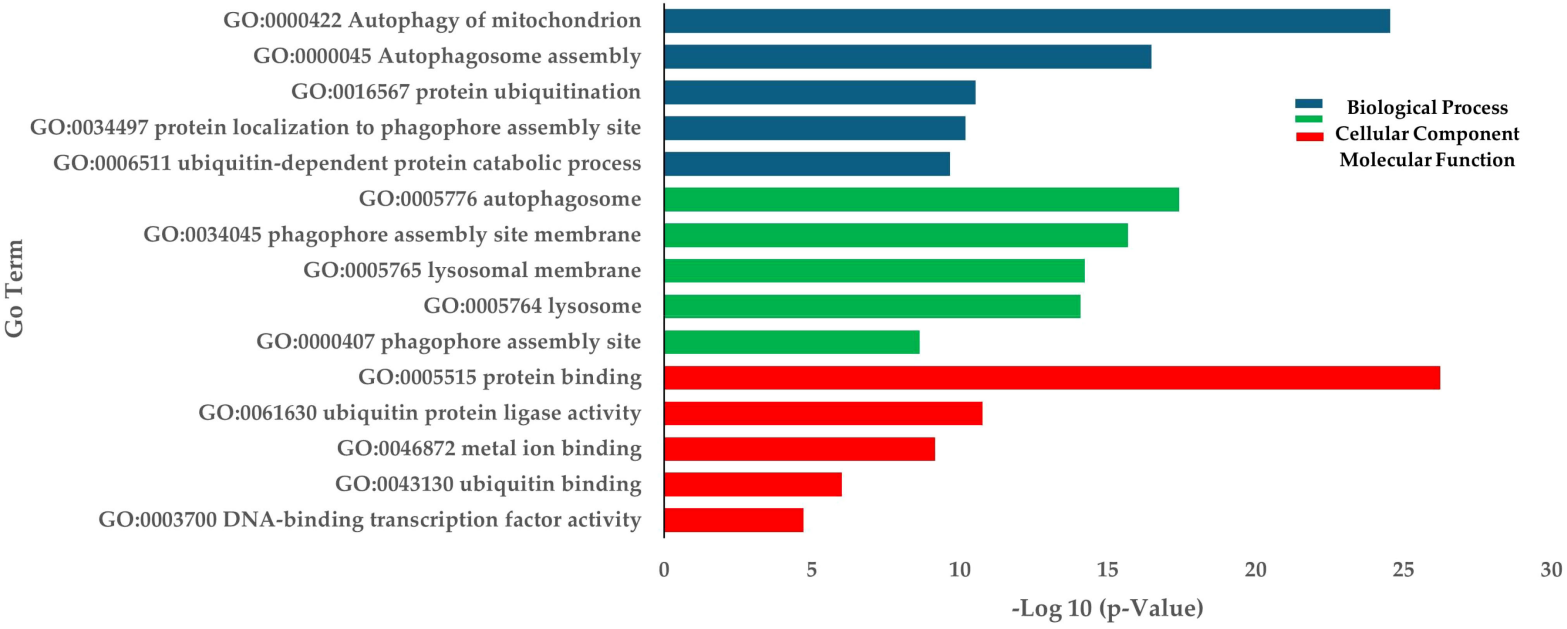
Enrichment of up-regulated DEGs in terms of BP (Biological process), CC (Cellular components), and MF (Molecular function) in HD group.

RNA sequencing was conducted using skeletal muscle samples collected 30 days after treatment from both the low density (LD) and high density (HD) groups. A total of 335,693,902 paired-end reads were generated from six cDNA libraries. The raw sequencing data have been deposited in the NCBI database under BioProject code PRJNA1189086. After filtering out adapter sequences and low-quality reads, 330,223,996 clean reads were retained for transcriptomic analysis (**Supplementary Table S3**). We identified 4,050 differentially expressed genes (DEGs) between the HD and LD groups, comprising 2,204 upregulated and 1,846 downregulated genes (**Supplementary Table S4**). GO and KEGG enrichment analyses were performed. For the upregulated DEGs, the most highly enriched Gene Ontology (GO) terms in biological processes (BP) included autophagy of mitochondria, autophagosome assembly, and protein ubiquitination (**Figure 3**). In the molecular function (MF) and cellular component (CC) categories, the most enriched GO terms were protein binding and autophagosome, respectively (**Figure 3**). KEGG pathway analysis revealed significant enrichment in autophagy, mitophagy, and lysosome pathways. Other interesting enriched KEGG pathways include Ubiquitin mediated proteolysis, mTOR signaling pathway, and FoxO signaling pathway (**Table 1**).

**Table 1:** KEGG pathways analysis of up-regulated DEGs in HD group.

KEGG Pathway	Gene Number	*P*-Value	Enrichment
Autophagy - animal	91	3.8 x 10^-29^	3.82
Mitophagy - animal	47	6.9 x 10^-12^	3.14
Lysosome	49	1.4 x 10^-11^	2.99
Phagosome	31	5.2 x 10^-3^	1.70
SNARE interactions in vesicular transport	11	8.5 x 10^-3^	2.63
Ubiquitin mediated proteolysis	28	1.5 x 10^-2^	1.62
mTOR signaling pathway	32	1.7 x 10^-2^	1.54
Adipocytokine signaling pathway	18	2.2 x 10^-2^	1.80
Proteasome	13	2.4 x 10^-2^	2.06
FoxO signaling pathway	10	3.9 x 10^-2^	2.18

**Figure 4:**
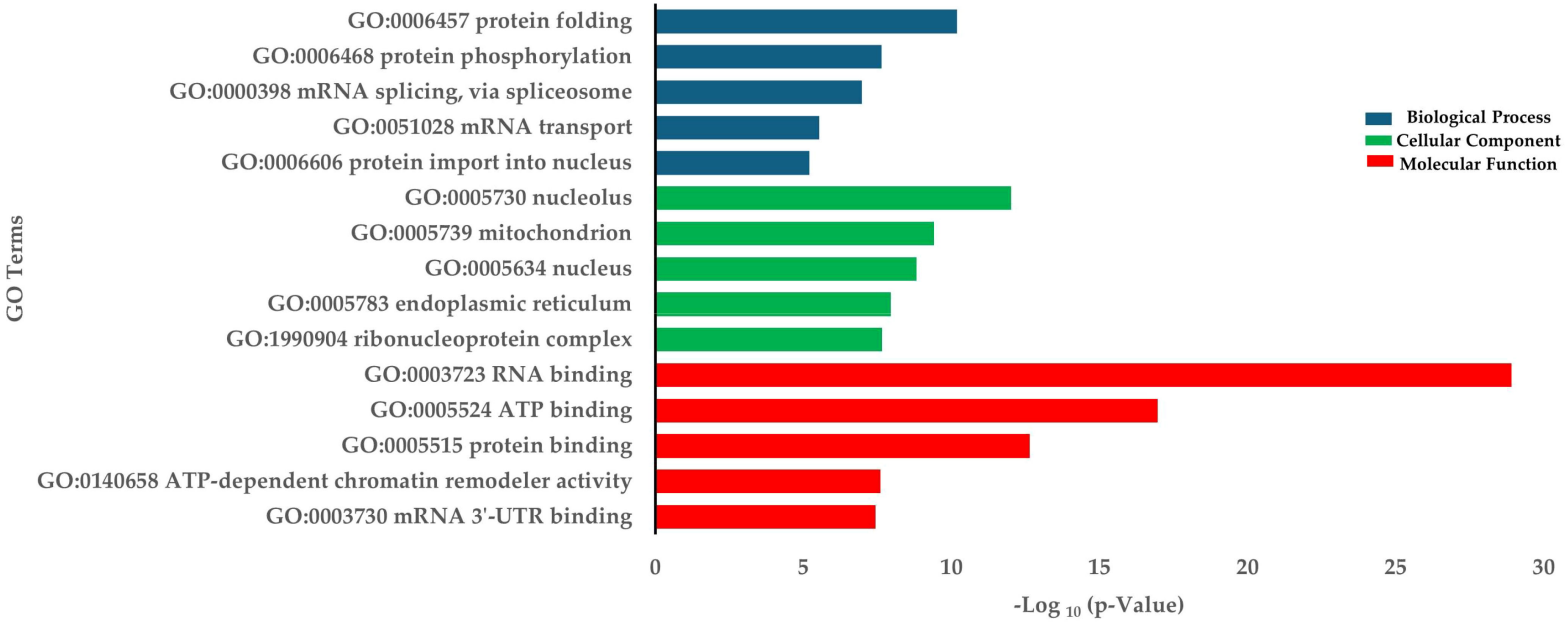
Enrichment of down-regulated DEGs in terms of BP (Biological process), CC (Cellular components), and MF (Molecular function) in HD group.

The downregulated DEGs were enriched in biological processes such as protein folding, protein phosphorylation, and mRNA splicing via the spliceosome (**Figure 4**). GO terms for down-regulated genes were associated with RNA binding (MF) and the nucleolus (CC) (**Figure 4**). KEGG pathway analysis indicated significant enrichment in spliceosome, nucleocytoplasmic transport, and mRNA surveillance pathways. Other interesting enriched KEGG pathways include ATP-dependent chromatin remodeling, Cysteine and methionine metabolism, and Biosynthesis of amino acids (**Table 2**).

**Table 2:** KEGG pathways analysis of down-regulated DEGs in HD group.

KEGG Pathway	Gene Number	*P*-Value	Enrichment
Spliceosome	49	5.5 x 10^-12^	3.07
Nucleocytoplasmic transport	39	1.2 x 10^-11^	3.56
mRNA surveillance pathway	30	1.5 x 10^-7^	3.05
ATP-dependent chromatin remodeling	33	5.3 x 10^-6^	2.43
Cysteine and methionine metabolism	19	8.2 x 10^-6^	3.44
Biosynthesis of amino acids	24	3.7 x 10^-5^	2.64
Insulin signaling pathway	37	5.4 x 10^-5^	2.06
Protein processing in endoplasmic reticulum	40	6.4 x 10^-5^	1.98
Lysine degradation	19	2.1 x 10^-4^	2.71
Arginine and proline metabolism	15	1.1 x 10^-3^	2.74

### 3.3 DNA methylation analysis of crowding stress

**Figure 5:**
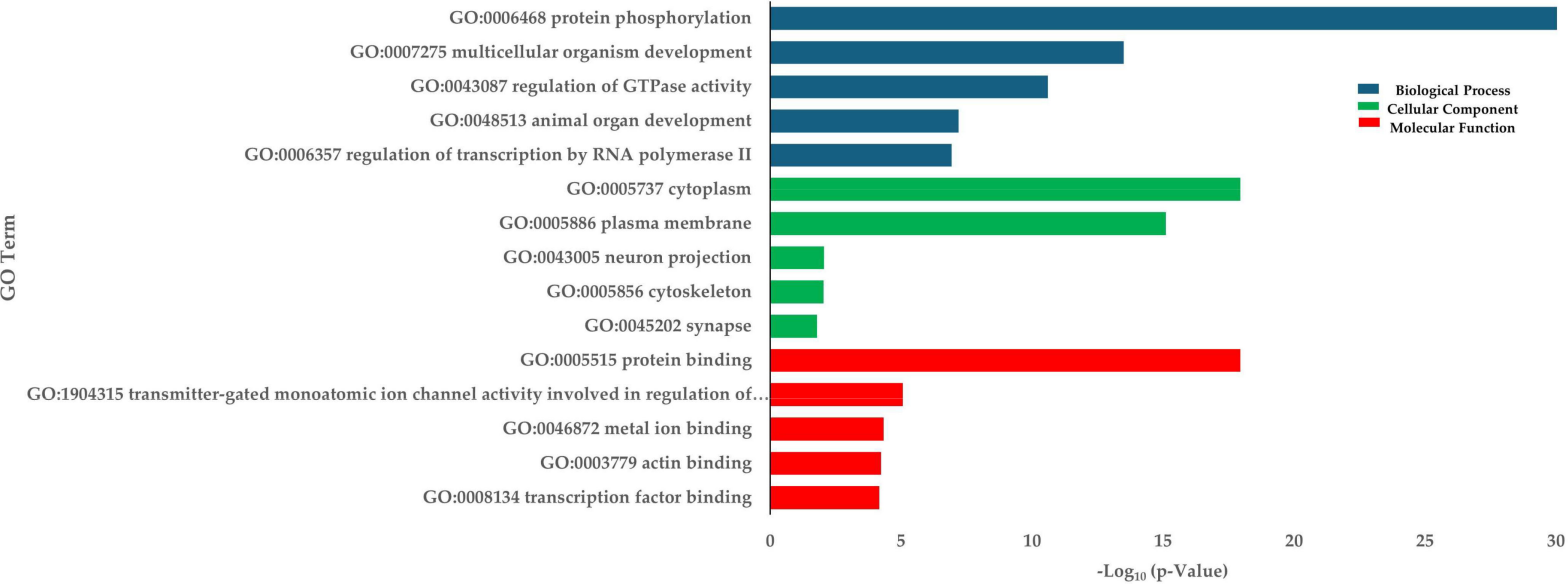
Enrichment of DMGs in terms of BP (Biological process), CC (Cellular components), and MF (Molecular function) in HD group.

To evaluate the impact of crowding stress on DNA methylation in Rainbow trout, bisulfite-treated DNA and genomic libraries were prepared from the skeletal muscle samples collected from the low-density (LD) and high-density (HD) groups at 30 days. A total of 455,885,911 reads were generated across six whole-genome bisulfite sequencing (WGBS) libraries. After filtering out low-quality reads, 455,077,237 clean reads were retained for methylation analysis. The bisulfite conversion was on average 99%, ensuring high data reliability (**Supplementary Table S5**). The average methylation level of CG was 85.3%. The rest of the cytosine methylations were found in the context CHG and CHH with 7.6%, and 7.1%, respectively (**Supplementary Table S6**). Differential DNA methylation analysis was conducted in the context of CG methylation. The analysis identified 76,749 differentially methylated regions (DMRs) in the rainbow trout genome, 41,178 regions up-methylated (**Supplementary Table S7**), and 35,571 down-methylated (**Supplementary Table S8**). The distribution of DMRs in the 32 rainbow trout chromosomes can be visualized in **Supplementary Table S9**. On average, 38% of DMRs are located at intergenic regions, 6% in promoter regions, 18% in exonic regions, and 38% in intronic regions. The annotation of these DMRs revealed 6,039 hyper-methylated genes and 5,633 hypomethylated genes. In total, 11,672 differentially methylated genes (DMGs) were identified. Enrichment analysis of DMGs highlighted biological processes (BPs) such as protein phosphorylation, multicellular organism development, and regulation of GTPase activity (**Figure 5**). Gene Ontology (GO) terms associated with DMGs were primarily linked to protein binding (MF) and the Cytoplasm (CC) (**Figure 5**). KEGG pathway analysis revealed significant enrichment in pathways such as glycerolipid metabolism, regulation of the actin cytoskeleton, and adherens junctions. Other interesting enriched KEGG pathways include Wnt signaling pathway, Focal adhesion, and Cytoskeleton in muscle cells (**Table 3**).

**Table 3:** KEGG pathways analysis of DMGs in HD group.

KEGG Pathway	Gene Number	*P*-Value	Enrichment
Glycerolipid metabolism	48	9.2 x 10^-5^	1.72
Regulation of actin cytoskeleton	164	1.3 x 10^-4^	1.30
Adherens junction	84	7.5 x 10^-4^	1.40
Wnt signaling pathway	125	8.6 x 10^-4^	1.30
Focal adhesion	143	9.8 x 10^-4^	1.27
Cytoskeleton in muscle cells	168	1.5 x 10^-3^	1.24
Progesterone-mediated oocyte maturation	61	2.3 x 10^-3^	1.43
Apelin signaling pathway	99	2.3 x 10^-3^	1.31
Tryptophan metabolism	27	4.7 x 10^-3^	1.70
Valine, leucine and isoleucine degradation	28	4.9 x 10^-3^	1.68

### 3.4 Integrative analysis of transcriptomic and epigenomic response of crowding

Integrative findings of RNA-seq and WGBS showed an overlap of 263 genes between 2,204 up-regulated expressed genes and 5,633 down-methylated genes. GO enrichment analysis of these genes, revealed that protein phosphorylation, regulation of transcription by RNA polymerase II, and endosome to lysosome transport were overrepresented in biological process (**Supplementary Table S10**). In cellular component and molecular function, cytoskeleton and protein binding were significantly enriched, respectively (**Supplementary Table S10**). The KEGG pathways analysis revealed that Autophagy, Mitophagy, Insulin signaling pathway were highly represented (**Table 4**). In the other hand, an overlap of 299 genes between 1,846 down-regulated expressed genes and 6,039 up-methylated genes were identified. GO enrichment analysis of these genes revealed that regulation of translation, auditory receptor cell development, and mRNA transport were highly represented (**Supplementary Table S11**). In cellular component and molecular function, cytoplasm and mRNA 3’-UTR binding were significantly enriched, respectively (**Supplementary Table S11**). The KEGG pathways analysis revealed that ATP-dependent chromatin remodeling, D-Amino acid metabolism, and Nucleocytoplasmic transport were overrepresented (**Table 4**).

**Table 4:** KEGG enrichment analysis of 263 up DEGs/down DMGs and 299 down DEGs/up DMGs in HD group.

Overlap	KEGG Pathway	*P*-Value	Gene list
Up DEG Down DMG	Autophagy - animal	6.7 x 10^-5^	*vps33a, zfyve1, supt20h, bnip3, rb1cc1, rab7, atg9b, atg9a, raf1, hif1a, ern1*
	Mitophagy - animal	3.5 x 10^-3^	*atg9b, bnip3, rab7, atg9a, hif1a, tbc1d15, zgc*
	Insulin signaling pathway	3.8 x 10^-2^	*socs2, prkag2, cip4, mnk2b, pde3b, prkag3raf1*
	Efferocytosis	8.0 x 10^-2^	*rab7, mapk14a, hif1a, fak1, vps33a, zgc*
Down DEG Up DMG	ATP-dep. chromatin remod.	6.1 x 10^-3^	*mta2, ep400, bptf, smarcc2, brd8, brg1, chd4, yeats4*
	D-Amino acid metabolism	9.7 x 10^-2^	*gls, gls2*
	Nucleocytoplasmic transport	9.8 x 10^-2^	*ranbp2, nup93, casc3, ahctf1, tnpo1*

**Figure 6:**
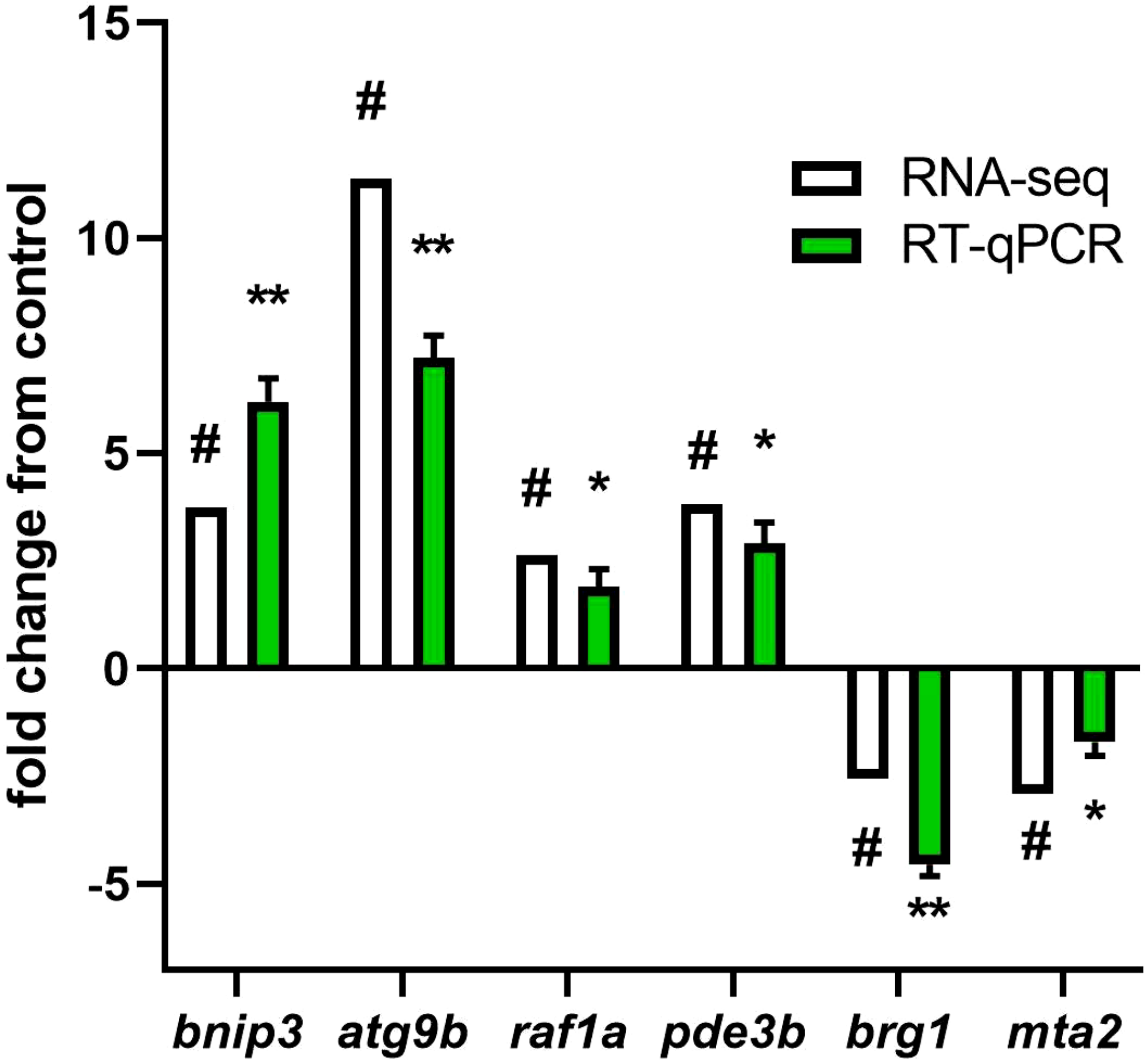
RT-qPCR validation of DEGs and DMGs of representative pathways. The genes selected for the RT-PCR validation were bnip3, atg9b, raf1a, pde3b, brg1, and mat2. For RNA-seq, in white, “#” indicates a log2 fold change ≥2.0 and FDR <0.05. For RT-qPCR, in green, relative expression was normalized against fau and actβ. The results are expressed as means ± standard errors (n=4). Differences are shown in *p < 0.05, and **p < 0.01.

For validation, we selected Bcl-2 interacting protein 3 (*bnip3*) and Autophagy Related 9B (*atg9b*) genes involved in autophagy and mitophagy. RAF proto-oncogene serine/threonine-protein kinase (*raf1a*) and Phosphodiesterase 3B (*pde3b*) genes, involved in insulin signaling pathway. Brahma-related gene 1 (*brg1*) and Metastasis-associated protein 2 (*mta2*) genes, involved in ATP-dependent chromatin remodeling. Our findings demonstrate a strong correlation (r = 0.9156) between gene expression levels obtained through RNA-seq and validated using qPCR. (**Figure 6**).

## 4 Discussion

Crowding stress significantly impacts on the welfare and growth performance of farmed fish. Fish, like other vertebrates, can experience psychological and ethological suffering under chronic stress, particularly in intensive aquaculture settings. Chronic stress, typically caused by prolonged exposure to environmental stressors, affects both their mental state and natural behaviors (12). Excessive stocking density may lead to suppressed growth, increased susceptibility to diseases, and higher mortality rates (37). In addition, crowding stress can enhance cannibalism in the aquaculture of some fish species due to resource competition, stress-induced aggression, and restricted movement (38). Our study aimed to assess the growth, physiological, and molecular impacts of stocking densities on rainbow trout, revealing those higher densities significantly impaired growth rates, physiological and oxidative status, as well as transcriptomic and epigenetic responses in skeletal muscle. Our results indicate that crowding stress causes a significant decrease in weight and condition factor at 30 days, accompanied by increased plasma levels of cortisol and creatine kinase. Numerous studies have shown that growth is negatively affected in various fish species when density levels exceed optimal thresholds, leading to significant declines in performance and overall health. According to FAO, intensive commercial farms for rainbow trout typically operate at maximum stocking density of 40–50 kg/m^3^ (5). For instance, in juvenile Rainbow trout, body weight and plasma cortisol levels were significantly altered at a stocking density of 30 kg/m³ after 45 days compared to fish reared at 10 kg/m³ (25). Similarly, a study conducted on the same species found that cortisol levels were significantly higher in fish reared at 40 kg/m³ and 80 kg/m³ compared to those kept at 10 kg/m³ for 30 days, although growth parameters were not quantified (39). In Atlantic salmon (*S. salar*), farming at 53 kg/m³ for 66 days led to decreased growth and increased plasma cortisol levels compared to 18 kg/m³ densities (40). Similar reports exist for non-salmonid species. In Fine flounder (*P. adspersus*), weight and condition factor were significantly reduced after seven weeks of crowding at 17 kg/m³ compared to fish reared at 7 kg/m³, with significant differences in cortisol levels observed only at week four (26). In Nile tilapia (*Oreochromis niloticus*), significant changes in weight and cortisol levels have also been reported with high-density farming (41). In summary, all these studies highlight a close relationship between crowding stress and elevated cortisol levels in plasma.

Increasing evidence suggests that elevated stocking densities can disrupt intracellular redox balance, leading to oxidative stress and cellular damage in various fish species (42). In our study, we determined that crowding stress induced oxidative damage in DNA and proteins in the skeletal muscle, without altering the antioxidant activity of catalase and superoxide dismutase (SOD). These observations are consistent with similar studies. For example, a recent study on Largemouth black bass (*Micropterus salmoides*) found that high farming densities (120 g/m³) induced oxidative stress and li-pid peroxidation in plasma and liver tissues after 90 days (43). Increasing Rainbow trout density to 25 kg/m³ for 70 days induced oxidative stress and up-regulated cytokine gene expression, although no variations were observed in catalase and SOD activity in the intestine, similar to our findings (44). Other studies suggest that crowding may increase antioxidant activity, indicating a possible response by the antioxidant system to stress (45). Oxidative damage associated with stress is not limited to crowding but also involves other stressors. For example, thermal stress has been shown to cause oxidation of proteins, nucleic acids, and lipids in the marine teleost red cusk-eel (*Genypterus chilensis*) (46). Interestingly, our research group recently described the effects of temperature stress in Rainbow trout, showing an increase in autophagy in skeletal muscle, but without linking it to oxidative damage (47). The relationship between stress and oxidative damage in fish skeletal muscle may be primarily regulated by cortisol and its non-genomic pathway. In Rainbow trout myotubes, cortisol has been shown to induce a rapid increase in plasma levels of reactive oxygen species (ROS), triggering a series of events associated with the ERK1/2 MAP kinase pathway (48).

Cortisol plays a central function in modulating both genomic and epigenomic responses to stress (49). It exerts its effects through genomic mechanisms by binding to glucocorticoid receptors, which act as transcription factors that directly regulate the expression of genes involved in stress responses, metabolism, and growth (50). Recent research has also shown that cortisol induces epigenomic changes, such as DNA methylation, which can influence long-term gene expression. These epigenetic changes may create a “stress memory” within the organism, meaning that fish exposed to chronic stress may have altered responses to subsequent stressors, potentially affecting growth, immune responses, and behavior (34). In the present study, we found that after 30 days of crowding stress, the expression of four thousand genes was altered, primarily associated with up-regulated pathways like autophagy, mitophagy, and the ubiquitin-proteasome system, and down-regulated pathways such as the spliceosome, nucleocytoplasmic transport, and mRNA surveillance. These results align with data published by our group, which described how crowding stress induces skeletal muscle protein degradation through upregulation of the UPS and autophagy pathways, while downregulating IGF-1 components during the stress response (25, 26). The involvement of cortisol in the regulation of gene expression related to protein catabolism in skeletal muscle has been demonstrated through various approaches (50). However, there is no evidence showing that changes in the expression of autophagy genes can be modulated by epigenomic remodeling. To explore this further, we examined the dynamics of DNA methylation in response to crowding stress using whole-genome bisulfite sequencing (WGBS). Methylation analysis revealed 76,748 differentially methylated regions (DMRs) in the Rainbow trout genome, with CG methylation being far more abundant than CHG and CHH methylation, which is consistent with other reports (51, 52). GC methylation was evenly distributed across all chromosomes, predominantly located in gene bodies and promoter regions near the transcription start site, similar to previous studies (53, 54). To assess the influence of DNA methylation on biological processes associated with crowding stress, we conducted a negative correlation analysis between differentially expressed genes (DEGs) and genes with altered methylation levels in their promoter regions or gene bodies (DMGs). We identified 263 genes showing a negative correlation between up-regulated expression and down-methylation, primarily related to autophagy, mitophagy, and insulin signaling pathways. Additionally, 299 genes showed a negative correlation between down-regulated expression and up-methylation, mainly linked to ATP-dependent chromatin remodeling, D-amino acid metabolism, and Nucleocytoplasmic transport. Few studies have explored the effects of stress on skeletal muscle in fish, particularly examining how methylation patterns and gene expression are differentially influenced. To our knowledge, no studies have analyzed the effects of crowding stress on genome methylation and epigenetic remodeling in teleosts. However, there are some interesting reports on multi-stressor and acute stress. A study on early life stressors, like cold shock and air exposure, showed that DNA methylation patterns and gene expression were altered in Atlantic salmon (*S. salar*), highlighting the role of environmental factors in shaping the methylome (55). Similarly, whole-genome bisulfite sequencing has been used to investigate DNA methylation changes in Atlantic salmon under acute (cold-shock during embryogenesis) and chronic stress (lack of tank enrichment during larval stages), revealing contrasting effects on DNA methylation linked to immune responses and gene expression (56). The same technical approach (WGBS) has been used to demonstrate differential methylation of sperm in Atlantic salmon (*S. salar*) in response to captivity, revealing how environmental factors influence gamete DNA methylation and contribute to transgenerational plasticity in this species (57). Our research group recently explored the effects of environmental factors on epigenomic and transcriptomic responses in Atlantic salmon during infection by sea lice (*Caligus rogercresseyi*) (58) and the effects of cortisol-mediated stress on transcriptomic and epigenomic responses in rainbow trout skeletal muscle (34). Both studies demonstrate a strong relationship between DNA methylation, gene expression, and the type of stress.

To validate our findings, we selected six genes that exhibited a negative correlation between methylation levels and gene expression, related to autophagy, the insulin pathway, and ATP-dependent chromatin remodeling. BCL2 interacting protein 3 (*bnip3*) is a gene involved in autophagy, mitophagy, and cell death (59). Overexpression of *bnip3* under high-density farming has been reported in Fine flounder (*P. adspersus*) (26) and Red claw crayfish (*Cherax quadricarinatus*) (60). Although no reports indicate changes in the methylation of this gene due to crowding stress, promoter region methylation of bnip3 has been described under hypoxic conditions in Rainbow trout (61). *Atg9b*, a crucial autophagy-related gene, plays a pivotal role in initiating autophagy by facilitating the formation and trafficking of autophagosomes. Unlike other autophagy-related genes, ATG9B is a transmembrane protein, making it essential for membrane delivery to expanding autophagosomes (62). Interestingly, its expression has been shown to regulate autophagy during immune responses in fish muscle cells challenged with the intracellular pathogen *P. salmonis* (63). Although there are no reports of methylation of this gene in aquatic organisms, its epigenetic regulation is essential for controlling autophagy in breast carcinoma, increasing the degradation of cellular proteins, similar to our observations (64). *Raf1*, also known as RAF proto-oncogene ser-ine/threonine-protein kinase gene, is a kinase that primarily functions as part of the MAPK/ERK signaling pathway, playing an essential role in regulating cell growth, differentiation, and survival. In the context of autophagy, raf1 has a dual role, as it can both promote and inhibit autophagic processes depending on cellular conditions and stimuli (65). Its expression has been upregulated as a mediator of temperature stress-induced autophagy in rainbow trout (47). Phosphodiesterase 3B (*pde3b*) is an enzyme that breaks down cyclic AMP (cAMP), a molecule that plays a key role in cell signaling, including autophagy regulation. In the context of autophagy, PDE3B’s activity indirectly influ-ences autophagic processes through its modulation of the cAMP/protein kinase A (PKA) pathway (66). To our knowledge, there are no previous reports about methylation of these genes in teleost under stress. Finally, we validated the downregulation of *brg1* and *mta2* involved in ATP-dependent chromatin remodeling. The Metastasis-Associated (*mta*) gene family, particularly *mta2*, is involved in chromatin remodeling and has a substantial role in regulating gene expression. The proteins encoded by *mta* genes are core components of the nucleosome remodeling and histone deacetylation (NuRD) complex, a multi-protein complex that modifies chromatin structure by both remodeling nucleosomes and deacetylating histones (67). *Brg1* (Brahma-related gene 1) is a critical ATPase and a central component of the SWI/SNF chromatin remodeling complex, which plays a crucial role in regulating chromatin structure and gene expression. This protein facilitates the ATP-dependent alteration of nucleosome positioning, making DNA more or less accessible for transcription and other nuclear processes (68). There are no studies that analyze its expression under stress conditions in fish, and even less its epigenetic regulation. Cortisol’s role in gene methylation is not fully understood, but studies in mammals show that glucocorticoids can alter DNA methylation through modulation in the expression of methyltransferases. DNA methylation occurs when DNA methyltransferases (DNMTs) add methyl groups to cytosine, creating 5-methylcytosine (5mC), which suppresses gene expression. In contrast, ten-eleven translocation (TET) proteins convert 5mC to 5-hydroxymethylcytosine (5hmC), promoting gene expression by leading to unmethylated cytosine (69, 70). Our future studies will investigate how cortisol and stress regulate differential gene methylation and expression, increasing the number of biological and technical replicates per treatment while addressing the failure to measure incidental variables that are essential for a broader understanding of our results.

## 5 Conclusion

This study highlights the significant impact of crowding stress on the growth and skeletal muscle development of juvenile rainbow trout in aquaculture. Crowding, a common chronic stressor, triggers the release of cortisol, redirecting energy from growth to stress response mechanisms, which ultimately impairs growth and muscle quality. The molecular analysis presented here reveals extensive physiological, oxidative, and epigenetic changes induced by crowding, including alterations in gene expression and DNA methylation. Notably, genes related to autophagy, mitophagy, and insulin signaling were found to be affected by crowding stress, while chromatin remodeling pathways were also implicated in the stress response. These findings provide novel insights into the molecular mechanisms underlying stress-induced growth impairments and emphasize the importance of optimizing farming conditions to improve fish welfare. By integrating transcriptomic and epigenetic data, this research paves the way for more sustainable aquaculture practices that can mitigate the detrimental effects of chronic stress on farmed fish. Future research should focus on investigating the mechanisms through which cortisol modulates differential gene methylation, increasing the number of biological and technical replicates per treatment.

## Data Availability

The data presented in the study are deposited in the NCBI database repository, accession number BioProject code PRJNA1189086.
